# Role of Oxygen and Its Radicals in Peripheral Nerve Regeneration: From Hypoxia to Physoxia to Hyperoxia

**DOI:** 10.3390/ijms25042030

**Published:** 2024-02-07

**Authors:** Dominik André-Lévigne, Rodrigue Pignel, Sylvain Boet, Vincent Jaquet, Daniel F. Kalbermatten, Srinivas Madduri

**Affiliations:** 1 Division of Plastic, Reconstructive and Aesthetic Surgery, Geneva University Hospitals, 1205 Geneva, Switzerland; 2 Subaquatic and Hyperbaric Medicine Unit, Division of Emergency Medicine, Department of Anesthesiology, Clinical Pharmacology, Intensive Care and Emergency Medicine, Geneva University Hospitals and Faculty of Medicine, University of Geneva, 1205 Geneva, Switzerland; 3 Department of Anesthesiology and Pain Medicine, The Ottawa Hospital, Ottawa, ON K1H 8L6, Canada; 4 Ottawa Hospital Research Institute, Clinical Epidemiology Program, Department of Innovation in Medical Education, University of Ottawa, Ottawa, ON K1H 8L6, Canada; 5 Institut du Savoir Montfort, Ottawa, ON K1K 0T2, Canada; 6 Department of Cell Physiology and Metabolism, University of Geneva, 1205 Geneva, Switzerland; 7 READS Unit, Faculty of Medicine, University of Geneva, 1205 Geneva, Switzerland; 8 Bioengineering and Neuroregeneration Laboratory, Department of Surgery, University of Geneva, 1205 Geneva, Switzerland

**Keywords:** nerve repair, nerve regeneration, axonal growth, sensory function, motor function

## Abstract

Oxygen is compulsory for mitochondrial function and energy supply, but it has numerous more nuanced roles. The different roles of oxygen in peripheral nerve regeneration range from energy supply, inflammation, phagocytosis, and oxidative cell destruction in the context of reperfusion injury to crucial redox signaling cascades that are necessary for effective axonal outgrowth. A fine balance between reactive oxygen species production and antioxidant activity draws the line between physiological and pathological nerve regeneration. There is compelling evidence that redox signaling mediated by the Nox family of nicotinamide adenine dinucleotide phosphate (NADPH) oxidases plays an important role in peripheral nerve regeneration. Further research is needed to better characterize the role of Nox in physiological and pathological circumstances, but the available data suggest that the modulation of Nox activity fosters great therapeutic potential. One of the promising approaches to enhance nerve regeneration by modulating the redox environment is hyperbaric oxygen therapy. In this review, we highlight the influence of various oxygenation states, i.e., hypoxia, physoxia, and hyperoxia, on peripheral nerve repair and regeneration. We summarize the currently available data and knowledge on the effectiveness of using hyperbaric oxygen therapy to treat nerve injuries and discuss future directions.

## 1. Introduction

Peripheral nerve injuries represent a substantial public health burden worldwide and are often associated with lifelong disabilities. Peripheral nerve damage can occur as a result of a variety of causes, such as trauma, infections, tumors, or autoimmune disorders. The severity and type of injury will determine the extent of damage to the nerve and the prognosis for recovery. The process of axonal outgrowth is complex and requires the coordinated activity of various cell types, including neurons and their axons; Schwann cells, which provide support and guidance for the growing nerve fibers; and macrophages, which help clear debris and provide a permissive environment for nerve regeneration. The process of nerve regeneration can take several weeks to several months. During this time, various factors can influence the success of nerve regeneration, such as the age and overall health of the patient, the location and extent of the injury, and the availability of supportive treatments [1].

When axonal continuity is disrupted, regeneration rates can reach 1–3 mm per day depending on the type and location of injury, whether a piece of nerve is missing, and whether the two ends are properly aligned. With microsurgical techniques, the outcomes of transected nerve injuries have significantly improved, but they are still often unsatisfactory. The longer the distance of nerve tissue that must be regenerated, the poorer the outcome, as the regenerative capability of neurons and the growth support of glial cells decrease. Although significant efforts have been made to find supportive therapeutic options to enhance regeneration capacities, there is still a lack of reliable and effective methods to improve outcomes in patients suffering from peripheral nerve injury [2].

Like all regenerative processes, extensive amounts of oxygen are needed as a source of energy in almost all processes of nerve regeneration. Biosynthesis, intracellular transportation, and cell division, as well as cell movement, rely on adenosine triphosphate (ATP), which is most efficiently synthesized in an oxygen-dependent manner in the mitochondria during oxidative phosphorylation [3]. However, oxygen and its radicals are likely to also play other key roles during nerve regeneration in both physiological and pathological conditions. Over the past few decades, the understanding of the role of oxygen in cell physiology has evolved from its long-recognized importance as an essential factor in oxidative metabolism and its germicide role in host defense to its recognition as an important player in cell signaling via reactive oxygen species (ROS) [4,5].

ROS are oxygen-derived small molecules that react with a wide range of factors, including other small inorganic compounds, carbohydrates, lipids, proteins, and nucleic acids [6]. It is generally accepted that ROS are major contributors of cellular and tissue damage during the aging process [7] by oxidizing both DNA and membranes, which leads to cell dysfunction and plays a role in neurodegeneration [8]. An imbalance between the production of ROS and their neutralization by the antioxidant cell defense system leads to oxidative stress, irreversibly damaging macromolecules. Oxidative stress has been repeatedly associated with peripheral nerve degeneration and associated disorders [9], and the antioxidant capabilities of Schwann cells are crucial to maintain oxidative balance within neurons [10].

However, ROS also have beneficial roles. The concerted production of large amounts of ROS by immune cells is of great importance for effective host defense and inflammatory activation [11]. ROS, in low concentrations, are involved in a myriad of physiological cell signaling pathways, referred to as redox signaling pathways [6]. Sies at al. suggested the term ‘oxidative eustress’ (in opposition to oxidative stress) to describe the physiological ROS levels at a nanomolar range [12]. Of particular importance for redox signaling, ROS are produced by the Nox family of nicotinamide adenine dinucleotide phosphate (NADPH) oxidases. The Nox family consists of membrane-bound complexes that transport electrons across biological membranes to reduce oxygen to ROS [6], mediating redox signaling through the direct reversible oxidation of proteins on cysteine or methionine residues [13,14]. The specific ROS that are produced; their amount, half-life, and diffusion distance; as well as the intracellular localization, dictate their biological outcomes [15]. There is increasing evidence that Nox-mediated ROS are crucial for regenerative processes, including skin wound repair [16,17,18]. It has been shown that Nox-derived ROS influence wound healing in zebrafish, including fin and skin innervation [19], neurite outgrowth in hippocampal neurons and cell lines [20], as well as whole-body regeneration in planarians [21].

The role of Nox-mediated ROS in peripheral nerve regeneration has been little explored, but recent data suggest that redox signaling plays a crucial role in key regenerative processes [22,23]. The anatomy of peripheral neurons with their long axonal and dendritic processes require compartmentalized signaling and the transportation of molecules and organelles across long distances, including during axonal development and regeneration after injury [24,25]. ROS are ideally suited to meet these signaling requirements, especially when considering that injury is followed by an inflammatory response surrounding the axon, thus increasing the local level of oxidants. However, the role of redox signaling and Nox in peripheral nerve regeneration is an emerging field of research, and the data on this topic are limited.

In this review, we discuss the influence of the oxygenation state, i.e., hypoxia, physoxia, and hyperoxia, and the repercussions of its relative changes on nerve regeneration, with a special focus on redox signaling. Here, we define physoxia as ‘normal’ oxygen levels, we define hypoxia as oxygen levels beneath the physiological oxygenation levels, representing an acute decrease in oxygen supply, and we define hyperoxia as an externally enhanced oxygen supply that can be achieved through hyperbaric oxygen therapy (HBOT). It is important to highlight that physiological oxygenation levels vary between tissues and that the oxygenation of peripheral nerve tissue is likely to vary depending on its location in the body. We are not aware of any study that has measured the physiological oxygenation levels specifically in peripheral nerves, but it is reported that the physiological oxygenation levels in normal tissues are maintained at 3–7% [26].

## 2. Role of Oxygen in Normal Nerve Function

Oxygen is critical for normal peripheral nerve function. Nerve cells require oxygen to generate energy in their mitochondria, which is essential for nerve impulse transmission and other cellular processes. Without adequate oxygen, nerve cells may not be able to produce enough ATP to support normal function. The extreme polarity of neuronal cells requires an effective mitochondrial transport system to allow for adequate energy supply all the way to the distal nerve ends [27]. Neurons, including peripheral neurons, contain high amounts of mitochondria and are high energy consumers [28]. In fact, mitochondria make up half of the cytoplasmic volume of neurons, and it has been reported that peripheral neurons under normal physiologic conditions require up to 4.7 billion ATP molecules per second in order to keep up their membrane potential over their large surface area [29]. In addition, oxygen is important for maintaining the health and function of the myelin sheath that surrounds nerve fibers. Schwann cells require oxygen to produce the lipids and proteins that constitute the myelin sheath. Of note, it has been suggested, based on molecular dynamics simulations, that stacked membranes have increased oxygen storage capacities, suggesting that myelin sheaths function as oxygen reservoirs [30].

Besides the role of oxygen as an energy supplier, an important physiological function of oxygen is derived from the production of ROS. Cellular Nox expression and the role of Nox-mediated redox signaling in the peripheral nervous system remain less understood as opposed to the better characterized central nervous system [31]. Several Nox isoforms are expressed at multiple peripheral nervous system sites [32,33]. Isoforms Nox1, Nox2, and Nox4 are expressed in dorsal root ganglion neurons [33,34,35], and rodent primary cultures of dorsal root ganglion neurons were shown to express Nox accessory proteins, such as p22phox and p47phox [36]. Puntambekar et al. showed that stimulating dorsal root ganglion cells with nerve growth factor (NGF) increases the expression of the Nox subunits Rac1 and gp91phox. They found that Rac1 was necessary for effective stimulation by NGF of the transient receptor potential channel TRPV1, which is essential in pain and heat sensation and aids in the maintenance of peripheral neuron integrity [37]. Indeed, Nox expression in the peripheral nervous system seems to be maintained at low basal levels under physiological conditions and upregulated during nerve regeneration after injury [23,31]. Nox-mediated redox levels are likely to be involved in both anti- and proapoptotic signaling cascades depending on the concentration of ROS produced. For instance, Nox-mediated ROS may mediate early apoptotic signaling in neuronal cell death in the central nervous system [38]. Of note, it has been shown that the NGF-deprivation-induced apoptosis of sympathetic neurons is Nox-dependent [39].

Of note, redox switches of Nrf2 and nuclear factor κB (NFκB), as well as sensory neuron ion transient receptor potential channels, have been reported to play roles in peripheral nerve function [23,40,41,42]. Gamper et al. conducted a review summarizing the redox-mediated regulation of ion channels in sensory neurons, highlighting the highly complicated interplay of redox signaling and ion channel excitability, including M channels, ATP-sensitive K^+^ channels, and many more [42].

It is conceivable that ROS play important roles in proper cell function in peripheral nerve tissue, but most available studies have focused on the negative impact of high concentrations of ROS, i.e., oxidative stress. Nerve-related disorders are associated with oxidative stress, as already demonstrated for diabetes-related and chemotherapy-induced peripheral neuropathies [43,44].

A fine balance between oxidative stress and antioxidants is necessary for effective cell function, particularly nerve function. When oxidant concentrations are too high, irreversible cellular damage occurs [45]. Intracellular oxidative stress is balanced by antioxidant enzymes and nonenzymatic scavengers. Nonenzymatic scavengers are of dietary origins, such as α-tocopherol (vitamin E), β-carotene, and ascorbate (vitamin C). In the peripheral nervous system, the main antioxidant enzymes are glutathione peroxidase (GPx), superoxide dismutase (SOD), and catalase, and the gene expression of these antioxidant enzymes is tightly regulated by the redox-sensitive transcription factor erythroid 2-related factor 2 (Nrf2) [10,46,47]. Besides its well-established effect on neurons by promoting their proliferation and survival when binding to its main target receptor tyrosine kinase A, NGF has also been associated with neuroprotective effects against oxidative stress. In fact, NGF protects against oxidative stress by increasing the expression of heme oxygenase-1 (HO-1), an enzyme that is responsible for the breakdown of heme, in a phosphatidylinositol 3-kinase-dependent manner [48]. In line with these findings, Dai et al. found that the systemic administration of NGF provided protection against colistin-induced peripheral neurotoxicity via the activation of the Akt and Nrf2/HO-1 pathways and the inhibition of oxidative stress [49].

Schwann cells are likely to play a prominent role in the antioxidant defense mechanisms of neurons as they express high levels of antioxidants. In an in vitro model of mild oxidative stress induced by adding H_2_O_2_ to dorsal root ganglion, Schwann cells expressed much higher levels of antioxidants than neurons [10].

As oxidative stress has been identified as a disease-inducing mechanism in neuropathies, antioxidant therapies have been suggested as potential solutions. However, they have largely failed to prove their effectiveness in clinical trials [50,51]. The lack of effectiveness of indiscriminate antioxidant therapy may be due to the fact that physiologic and pathologic ROS sources are affected simultaneously. The current research now focuses on specifically targeting disease-related ROS sources [52].

## 3. Role of Oxygen and Its Radicals in Nerve Regeneration after Injury under Physiological (Physoxic) Conditions

Nerve regeneration involves a complex series of events, including the growth of new axons, the formation of new myelin sheaths, and the re-establishment of functional connections between nerve cells. Traumatic tissue damage is inherently associated with local ischemia as vessels are disrupted at the injury site. This local ischemia triggers hypoxic signaling cascades, inducing neoangiogenesis and inflammation, which are essential for effective regeneration. After tissue damage and throughout the physiological regenerative process, the local oxygen levels progressively increase as neoangiogenesis occurs and energy demands rise.

Other than being the main source of ATP, which is crucial for the whole regenerative process, the mitochondria play other primordial roles during peripheral nerve regeneration [53]. They are crucial for maintaining membrane potentials and for calcium and iron homeostasis [54,55]. The effective transport of mitochondria toward the injury site of the axon is needed for growth cone formation [56]. In fact, the depletion of the mitochondria-anchoring protein syntaphilin (SNPH), which is mainly present in mature neurons, led to accelerated axonal regrowth in a sciatic nerve crush model [57]. Mitochondrial dysfunction is associated with increased oxidative stress and causes a variety of neuropathies and impaired nerve regeneration. Mitochondrial dysfunction can be caused by genetic disorders, toxins, metabolic disorders, age, chronic inflammation, or nutritional deficiencies [58].

We describe the role of oxygen and its radicals in nerve regeneration after traumatic injury under physiologic conditions where systemic and regional oxygenation are normal.

### 3.1. Distal Axon Degeneration and Schwann Cell Function

Following an insult leading to axon discontinuity, macrophages and Schwann cells become activated and start removing myelin debris and producing chemokines, inflammatory cytokines, proteolytic enzymes, and growth factors needed for axonal regeneration [59]. In this initial phase, injured axons experience an inflammation-dependent and extremely oxidative environment, mostly due to macrophage-derived Nox2 activity that contributes to growth cone collapse and retraction, known as Wallerian degeneration, which occurs via the ROS-dependent oxidation of actin [60,61]. Schwann cells lose their characteristic of myelinating axons and shift into the state of developmental promyelinating cells. These recharacterized Schwann cells then align in what is known as ‘bands of Büngner’ and guide newly regrowing axons to their destination [59].

The interaction between neuronal cells and adjacent cell populations, namely Schwann cells and macrophages, is primordial for effectively guided axonal outgrowth. Recent data point towards Schwann cells and Schwann cell–axon crosstalk having substantial roles in axon viability and function, including controlling redox levels in neuronal mature tissues [62,63,64], energy substrate transfer from Schwann cells to axons during periods of high energy demand, and ROS scavenging by Schwann cells [10,65]. Redox signaling is likely to also play a role in Schwann cells’ shift in phenotype during Wallerian degeneration and the formation of Büngner bands, but specific data on this topic are scarce. Injury-induced dysmyelination processes in peripheral nerves appear to be Nox4-dependent, as indicated by a decreased degradation of peripheral myelin proteins and by ameliorated structural changes in the injured sciatic nerve in Nox4-deficient mice [66]. Of particular interest is the role of Schwann cells as the ‘guardians’ of the redox environment during peripheral nerve repair. Lv et al. showed that Nrf2, which acts as a master regulator of antioxidant cell defense, is downregulated after peripheral nerve damage, leading to increased oxidative stress. They found that deleting Nrf2 led to more effective dysmyelination but an impaired myelination and the redifferentiation of Schwann cells [47]. These results suggest that a temporary inactivation of the antioxidant system of Schwann cells takes place after nerve injury, giving rise to an oxidizing environment promoting Wallerian degeneration.

**Figure 1:**
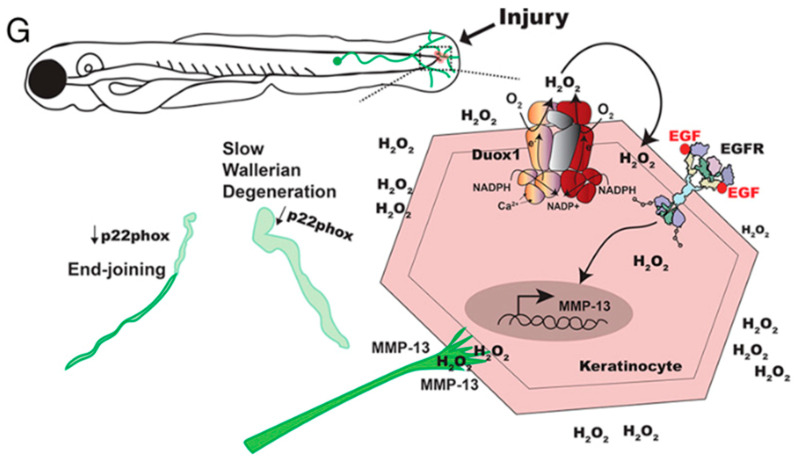
Zebrafish time-lapse imaging model of epithelial reinnervation after skin injury shows Nox mediation in skin de- and regeneration. It is shown that H_2_O_2_ leads to EGFR oxidation and MMP-13 activation, mediating axon growth within the epidermis. In fact, the keratinocyte-specific oxidation of EGFR serves as an attractive cue for regenerating sensory peripheral axons in the dermis. Loss of the neuron-intrinsic Nox1-4 subunit p22Phox slows Wallerian degeneration and promotes axonal fusion, showing a dual role of Nox-controlled H_2_O_2_ in skin de- and reinnervation. Reprinted from Cadiz Diaz et al. [67].

In a zebrafish time-lapse imaging model of epithelial reinnervation during wound repair, Cadiz Diaz et al. demonstrated that p22phox, a subunit required for Nox1-4 activation, promotes Wallerian degeneration and axonal fusion. They also show that cutaneous axon regeneration following injury relies on keratinocyte and neuron-specific H_2_O_2_ signaling. DUOX1-derived H_2_O_2_ leads to the redox activation of epidermal growth factor receptor (EGFR) in keratinocytes and promotes axonal outgrowth in the epidermis, indicating that EGFR oxidation by NADPH oxidase is critical for epidermal reinnervation. Hence, the authors were able to show that Nox-mediated H_2_O_2_ is implicated in both the de- and regeneration of sensory peripheral nerves after skin injury (Figure 1) [67].

### 3.2. ROS and Redox Signaling during Inflammation

The central role of ROS production during inflammation is well established. The isoform Nox2 plays a predominant role in early macrophage function and is responsible for producing large amounts of O_2_^•^ and H_2_O_2_ in what is called the respiratory burst [11,68]. Inside phagosomes, high concentrations of ROS create an oxidative environment for phagocytosed debris, leading to lipid peroxidation and the oxidation of amino acids [69]. Mutations leading to a defective phagocyte Nox2 system lead to chronic granulomatous disease, which is characterized by impaired wound repair and an increased susceptibility to infection [70,71].

Apart from their role in immune defense, ROS are central signaling molecules modulating the inflammatory response. Nox-mediated redox modifications of signaling proteins are likely mechanisms by which redox signaling plays a role in peripheral nerve regeneration. ROS oxidize specific cysteine residues and induce structural and functional changes in various proteins, including kinases, phosphatases, ion channels, and transcription factors [72]. Depending on their concentrations and via NFκB signaling cascades, ROS can either induce a pro-inflammatory control loop that promotes debris removal or an anti-inflammatory control loop that inhibits an exacerbated harmful inflammatory response [73]. Other compelling examples for the role of ROS in the regulation of inflammation are that low concentrations induce neutrophil chemotaxis [74], and the overexpression of the antioxidant thioredoxin suppresses leukocyte recruitment [75]. Furthermore, ROS modulate the expression of leukocyte–endothelial adhesion molecules [76] and induce the spread of macrophages via extracellular signal-regulated kinases [77]. In addition, monocytes are activated when they adhere to the extracellular matrix (ECM) by their specific integrin receptors. This adhesion can be induced by ROS in vitro [78].

**Figure 2:**
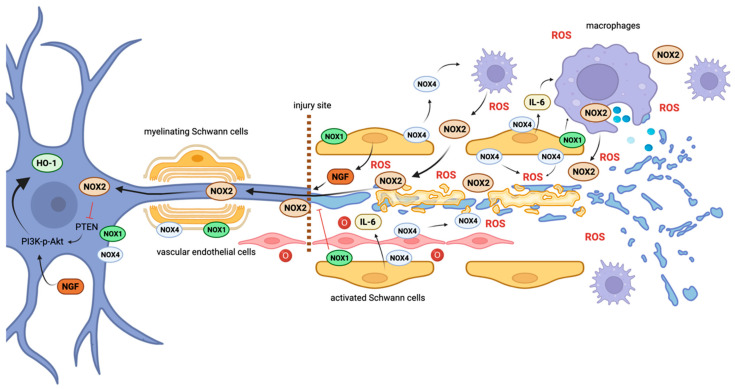
Schematic view of suggested key redox signaling processes in nerve regeneration. Inflammation during Wallerian degeneration is associated with an extremely oxidative environment induced by macrophages and Schwann cells. Large amounts of Nox2 are produced by invading macrophages. In addition, endothelium-derived exosomes contain Nox4 and produce ROS. Nerve growth factor induces neurite outgrowth in a ROS-dependent manner. Nox2 localizes in growth cones modulating neurite outgrowth by oxidizing the actin cytoskeleton and changing its polymerization state, while Nox1 seems to have a suppressive effect on neurite outgrowth. Nox2-containing exosomes released by macrophages are internalized by neuronal cells and transported to the cell body, causing oxidation-induced inactivation of PTEN, which enables activation of PI3K-p-Akt signaling and leads to axonal outgrowth and elongation.

Tumor necrosis factor alpha (TNF-α) and interleukin-6 (IL-6) induce neutrophil and macrophage migration and facilitate phagocytosis, and their biosynthesis has been shown to be ROS-inducible [79]. IL-6 is an important regulator in pro-inflammatory signaling in Schwann cells during peripheral nerve regeneration [80]. In the central nervous system, it has been reported that the expression of Nox4 in human microglia leads to IL-6 expression [81] and that the interplay between antioxidant systems and Nox2-mediated redox signaling is responsible for microglia activation [82]. Indeed, phagocytes (the main source of Nox2-derived ROS) could be recruited through Nox4-dependent IL-6 expression, similar to what has been shown in human microglia and non-small-cell lung cancer cells [81,83]. Schwann cells express Nox1 and Nox4 and probably no (or very little) Nox2 [23,32,84]. Extracellular Nox4-mediated ROS expression could lead to Nox2-expressing macrophage recruitment to the perineurial space [32]. In our laboratory, we found that a Nox4 deficiency leads to a decreased expression of Nox2 during cutaneous wound repair, implying that Nox4 has a role in phagocytic cell recruitment [85]. Indeed, a feed-forward loop leading to oxidative stress in the peripheral nervous system has been suggested between Schwann cell Nox1/Nox4 expression and macrophage Nox2 expression (Figure 2) [23].

Of note, this Nox4-dependent recruitment of NOX2-expressing phagocytes has also been reported to be implicated in the pathophysiology of diabetic peripheral neuropathy, which is associated with a pathological redox environment. Eid at al. showed that Nox4 inhibition decreases ROS production in Schwann cells and peripheral nerves and reverses the diabetes-induced alteration of peripheral nerve myelinization in a diabetic mouse model [84].

### 3.3. Retrograde Injury Signaling and Axonal Regeneration

Before transcriptional changes can occur in the injured neuron, a retrograde injury signal must be sent to the nucleus [1]. Traditionally, oxidative stress has been seen as neurotoxic, and ROS have been associated with neurite degeneration and the induction of cell death in the central nervous system [9]. However, there is a growing consensus that ROS can also play important beneficial roles in neuroregeneration. For instance, proliferative neural stem cells have high endogenous ROS levels that regulate self-renewal and neurogenesis in a PI3K-p-Akt-dependent manner [86]. Peripheral neurite outgrowth has been reported to be ROS-dependent in sensory neurons in a mouse sciatic nerve regenerative model [22]. Of note, the same authors demonstrate that macrophage-derived active Nox2 complexes are secreted via exosomes, incorporated into the endosomes of dorsal root ganglia, and are required for neurite outgrowth. These macrophage-derived, internalized Nox2-carrying exosomes are retrogradely transported into the cell body through the axonal transport system and have been visualized at 20 mm from the lesion site at 6 h after injury. Once it arrives at the cell body, Nox2 causes the oxidation-induced inactivation of PTEN, which enables the activation of PI3K-p-Akt signaling and leads to dorsal root ganglion axonal outgrowth and regenerative reprogramming after nerve injury [22]. In line with these results, De Virgiliis et al. demonstrated that the in vivo activation of neuronal Nox2 promotes axonal regeneration and the partial restoration of sensory nerve function after spinal cord injury [87].

There is convincing evidence that physiological Nox-derived ROS levels are required for growth cone formation. However, different Nox isoforms may have opposing effects on neurite outgrowth, and further research is needed to better characterize the role of Nox-mediated redox signaling during the development of growth cones [23,88]. NGF induces neurite outgrowth in cultured neurons in a H_2_O_2_-dependent manner [89]. Of note, increased Nox activity is required for Rac1-mediated neurite outgrowth in PC12 cell culture, which is further accelerated by the exogenous addition of H_2_O_2_ [90]. Ibi et al. found that PC12 cells express Nox1 at rates that are 10 times higher than Nox2 and do not express Nox4. When stimulated with NGF, PC12 cells’ expressions of Nox1 and Nox2 mRNA levels are increased. Nox1 level increases were associated with increased levels of intracellular O_2_^•^. However, cells depleted of Nox1 via a stable expression of ribozymes targeted for Nox1 mRNA showed significantly enhanced axonal outgrowth, suggesting that Nox1 negatively regulates neurite outgrowth [91]. Munnamalai et al. found that Nox2 protein is localized in growth cones and suggested that it may modulate neurite outgrowth by oxidizing the actin cytoskeleton and changing its polymerization state [92].

Effective axonal regeneration requires functioning Schwann cell–axon crosstalk [63,93]. Schwann cells are known to provide energy substrate transfer to axons during periods of high energy demand, but they are also able of acting as ROS scavengers, reducing oxidative stress in the axon [10,65]. The concept that Nox are transferred between cells via exosomes has gained increasing interest recently [94,95,96,97], and data suggest that exosomes are metabolically active and generate ROS. For instance, studies report that endothelium-derived exosomes contain Nox4 and produce Nox-dependent ROS [94,98,99]. Indeed, it is conceivable that redox signaling via Nox-containing exosomes between Schwann cells and axons plays a role in nerve regeneration (Figure 1) [23].

Inflammation during Wallerian degeneration is associated with an extremely oxidative environment induced by macrophages and Schwann cells. Large amounts of Nox2 are produced by invading macrophages. In addition, endothelium-derived exosomes contain Nox4 and produce ROS. Nerve growth factor induces neurite outgrowth in an ROS-dependent manner. Nox2 localizes in growth cones, modulating neurite outgrowth by oxidizing the actin cytoskeleton and changing its polymerization state, while Nox1 seems to have a suppressive effect on neurite outgrowth. Nox2-containing exosomes released by macrophages are internalized by neuronal cells and transported to the cell body, causing the oxidation-induced inactivation of PTEN, which enables the activation of PI3K-p-Akt signaling and leads to axonal outgrowth and elongation.

## 4. Repercussions of Ischemia/Hypoxia on Nerve Regeneration and Redox Signaling

Traumatic nerve injury leads to the disruption of the blood supply, resulting in a decrease in the oxygen supply in the nerve and surrounding tissue. Ischemia plays an important role in tissue regeneration in general and also in nerve regeneration. The initial appearance of local acute ischemia leads to signaling cascades, promoting neovascularization, inflammation, and cell proliferation [100]. The induction of hypoxia-inducible factor 1α (HIF1α) by deleting or pharmacologically inhibiting prolyl hydroxylase domain proteins, mimicking systemic hypoxia, enhances axonal regeneration and functional recovery following peripheral nerve injury [101]. In line with these findings, a positive effect of repeated brief ischemia in sciatic nerve transection injury has been found in a rat model and attributed to increased levels of vascular endothelial growth factor (VEGF) and Schwann cell proliferation in the ischemic environment [102] (Table 1).

Kim et al. reported that Nox-derived ROS play important roles in VEGF-mediated angiogenesis. They suggested that H_2_O_2_ released by Nox4 leads to the enhancement of VEGF receptor 2 (VEGFR2) signaling via Nox2 activation in a Nox4/Nox2/pSer36-p66Shc feed-forward loop, providing yet another compelling example of the interplay between Nox4 and Nox2 [103].

### 4.1. Prolonged Ischemia

Depending on the severity of the injury and the speed of blood flow restoration, prolonged ischemia might occur. Prolonged ischemia has several negative effects on the regenerative process, including energy deprivation and inflammatory processes. Nerve tissue is known to be particularly sensitive to prolonged ischemia and oxygen and nutrient depletion, leading to conduction failure and, eventually, fiber degeneration [104,105]. In a rat model of severe sciatic ischemia, it has been shown that 1 and 3 h of ischemia lead to conduction failure, with a recovery of less than 10% after reperfusion following 3 h of ischemia [104].

The degree of nerve damage and the onset of conduction failure depend on the specific nerve involved, the presence of collateral blood supply, and the underlying health of the individual. For instance, diabetes leads to morphological vulnerability and ischemia and reperfusion in a model of sciatic–tibial nerve conduction. In fact, diabetic nerves showed a delayed recovery of ischemic conduction failure after brief ischemia compared to controls [106]. In line with these findings, we found in a model of severe limb ischemia in rats that hypoglycemia increases the susceptibility to ischemic limb necrosis [107]. Interestingly, it has been suggested that age has a protective role in nerve resistance to ischemic conduction failure as it has been shown that rat peripheral nerves are progressively more resistant to ischemic–anoxic conduction failure with increasing age. The nerve lactate response to anoxia is higher in young rats than in older animals, and it has been suggested that a progressive decline in energy requirements with age is the mechanism behind this resistance to ischemic conduction failure in older individuals [108].

Of note, it has been reported that mitochondrial Nox4 in renal cells can act as an energetic sensor whose activity is directly regulated by ATP [109]. In fact, Nox4 could play an important role in cell survival during critical ischemia as it mediates autophagy in response to energy stress. It has been shown in cardiomyocytes that Nox4 stimulates the protein kinase RNA-activated-like ER kinase signaling pathway in response to energy stress-inducing autophagy [110]. Schwann cell autophagy plays an important role during nerve regeneration as it is a critical step in Wallerian degeneration and is required for effective Schwann cell reorganization [111]. With oxygen being their principal substrate, all members of the Nox family are strictly oxygen-dependent, and oxygen is the rate-limiting factor in Nox2-mediated ROS production during the respiratory burst of wound repair [112,113]. It is therefore plausible that ischemia has major repercussions on Nox activity.

### 4.2. Effects of Ischemia-Reperfusion after Injury

It is well established that when reperfusion occurs after prolonged ischemia, oxidative stress in endothelial cells leads to endoneurial edema, further aggravating fiber degeneration [114,115]. A severe inflammatory response then takes place, typically confined to a few days after reperfusion [116,117]. The molecular and cellular mechanisms of the unique injury response that results when ischemic tissues are reperfused has been the subject of intensive research [118,119,120]. In the early 1980s, ROS were first suggested as potential mediators of reperfusion injury [121], and Nox have been found to have central roles as mediators of reperfusion injury for a variety of tissues [121]. It has been hypothesized that Nox enzymes contribute to reperfusion injury, largely based on the fact that an increased expression and/or activity of Nox in post-ischemic tissue has been observed in a variety of tissues including the intestine, lung, heart, eye, brain, stomach, liver, kidney, and testes [122,123,124,125,126,127,128]. The pharmacological inhibition of Nox activity or the genetic inhibition/deletion of Nox activity/protein leads to an attenuation of reperfusion-induced injury response and/or reduced ROS production [121,125,129,130,131,132,133]. Of note, the inhibition and deletion of Nox have emerged as neuroprotective effects after ischemia-reperfusion in brain tissue as less intercellular adhesion molecule-1 upregulation and less neutrophil infiltration were found in Nox2-deficient mice and mice treated with the antioxidant apocynin following reperfusion [129].

In a model of myocardial ischemia-reperfusion injury, 30 min of ischemia followed by 24 h of reperfusion resulted in a significant decrease in the size of myocardial infarct in Nox1-, Nox2-, and Nox1/Nox2-deficient mice, but not in Nox4-deficient mice. Interestingly, no protection was observed in a model of chronic ischemia, strengthening the notion that Nox1- and Nox2-mediated oxidative damage occur during reperfusion [134]. In a study on reperfusion injury in rat brains, the findings suggested that Nox2 may not contribute to the early burst of reperfusion-related ROS generation, but is rather an important source of ROS generation during prolonged reperfusion [135]. These data from other organs suggest that Nox have a major role in reperfusion injury in peripheral nerves. However, specific data are still largely lacking.

**Table 1:** Repercussions of ischemia/hypoxia on nerve regeneration and redox signaling.

Oxygenation State	Observed Phenomenon	Reported Nox-Related Mechanism	Reference
Acute ischemia	Neoangiogenesis, inflammation, cell proliferation. Repeated brief ischemia stimulates nerve regeneration and increases levels of VEGF and Schwann cell proliferation.	Nox4/Nox2/pSer36-p66Shc feed-forward loop leading to VEGFR2 activation.	[100,101,102,103]
Prolonged ischemia	Energy depletion, conduction failure, necrosis.	NOX4 acts as an energy sensor, inducing autophagy and cell survival via the protein kinase RNA-activated-like ER kinase signaling pathway.	[104,105,110]
	Diabetes increases susceptibility to ischemic conduction failure and necrosis.	ROS overexpression in diabetic nerves is well established, and specific ROS sources are unknown.	[106,107,136]
Ischemia-reperfusion	Endoneuronal edema, fiber degeneration.	Excessive oxidative stress established as main pathomechanism.	[114,115,134,135]
		Increased expression/activity of Nox1 and Nox2, but not Nox4 in myocardial reperfusion.	[122,123,124,125,126,127,128,134]
		Neuroprotective effect of inhibition of Nox in reperfusion injury.	[121,125,129,130,131,132,133]

## 5. Effects of Hyperbaric Oxygen on Peripheral Nerve Regeneration

Considering the above-mentioned crucial oxygen-dependent mechanisms needed for effective nerve regeneration, it is conceivable that increasing blood oxygen concentration levels has major repercussions on neuroregeneration. In traumatic injuries where blood flow can be improved surgically, it should be carried out in a timely fashion to limit ischemic damage, as the nerve tissue is particularly vulnerable. However, nerve injury is inherently associated with some degree of local reduction in blood flow and remains inaccessible to surgical repair. Hence, the only way to increase the locally available oxygen supply is to increase the oxygen concentration in the remaining bloodstream, potentially leading to a more effective diffusion of oxygen to the injury site. Hemoglobin-bound oxygen availability is already at near-saturation levels under normal conditions and therefore cannot be significantly enhanced.

Various therapy options have been suggested to increase the tissue oxygen supply, including topical oxygen therapy (TOT), where tissues are directly exposed to 100% oxygen. The efficacy of TOT remains controversial as oxygen penetration at the air–liquid interface is very limited, although there is some evidence that TOT is effective in promoting skin wound regeneration [137]. Contrary to skin ulcers, the target tissue in peripheral nerve injuries lies deep in the body, and TOT is therefore unlikely to improve peripheral nerve regeneration. An effective tool to increase tissue oxygenation levels in deeper-lying tissues is HBOT, as it allows for an increase in freely dissolved oxygen in the bloodstream, thereby increasing systemic oxygen availability. The treatment involves placing a patient in a sealed chamber and elevating its pressure several-fold above the ambient air pressure while the patient breathes 100% oxygen. It has been repeatedly reported to have a pro-regenerative effect in a variety of tissues, but the exact mechanisms of action are still poorly understood, thus resulting in inconsistent treatment protocols and vague indications [138,139,140]. There is some evidence that HBOT has a beneficial effect on nerve regeneration, but it has not yet gained widespread acceptance in the treatment of nerve trauma patients [141].

Data on the effects of HBOT on Nox-derived redox signaling during peripheral nerve regeneration are largely lacking, and available findings may vary depending on the specific experimental conditions, animal models, and types of injuries studied. As described above, the roles of oxygen, its radicals, and, more specifically, Nox-mediated redox signaling in peripheral nerve regeneration are complex as they are involved in both beneficial and detrimental processes.

We suggest that HBOT influences the redox environment in the context of peripheral nerve regeneration in the following ways (Table 2):

1. It induces the antioxidant response;
2. It limits detrimental Nox-mediated ROS production during reperfusion-injury and the modulation of the inflammatory response;
3. It induces relative hypoxia-inducing neoangiogenesis;
4. It has a pro-regenerative effect through redox-mediated growth factor activation.

### 5.1. Induction of Antioxidants by HBOT

One of the most compelling hypotheses of the mechanism behind the beneficial effect of HBOT on reperfusion injury is the protective effect of the treatment through an antioxidant response that hyperbaric oxygen itself produces via the induction of antioxidants. This has been described as the ‘hyperbaric oxygen paradox’ in reperfusion injury [139]. Oxidative stress was found to be caused by preconditioning with hyperbaric oxygen and hyperoxia-induced tolerance against spinal cord ischemia in rabbits [142] by the upregulation of antioxidant enzymes [143] and was found to promote the production of glutathione, the principal non-enzymatic body defense against ROS [144]. Similar to these findings, HBOT promoted the production of enzymatic antioxidants, such as glutathione peroxidase and catalase, in a model of necrotizing pancreatitis [145]. A recent study investigating the neuroprotective effects of HBOT on neuronal death induced by sciatic nerve transection in a rat model revealed that the malondialdehyde (a product of lipid peroxidation) levels were significantly decreased in the HBOT group, while the antioxidant activities of superoxide dismutase and catalase were significantly increased [146]. Based on these findings, the authors concluded that both pre- and post-HBOT have neuroprotective effects against sciatic nerve transection-induced degeneration. Other examples of the protective antioxidant effects of HBOT are the increase in the production of IL-10, the inhibition of inducible and neuronal nitric oxide synthase, and the upregulation of key antioxidant and anti-apoptotic factors, such as BCL-2, heme oxygenase-1, and heat-shock proteins 70 and 72 [147,148,149,150,151,152]. In a rat subarachnoid hemorrhage model, HBOT decreased Nox2 expression and activity and the level of oxidative stress at 24 h after hemorrhage, thereby reducing neuronal injury and improving functional performance [153].

As mentioned previously, NGF is associated with neuroprotective effects against oxidative stress as it increases the expression of HO-1 [48]. In a model of traumatic brain injury, HBOT promoted the expression of neurotrophic factors such as NGF, brain-derived neurotrophic factor (BDNF), glial cell line-derived neurotrophic factor (GDNF), and neurotrophin-3 (NT-3) in vivo [154]. Hence, it is conceivable that HBOT confers a neuroprotective effect through the induction of neurotrophic factors, such as NGF, which, in turn, induces antioxidative mechanisms.

### 5.2. HBOT, Reperfusion Injury, and Excessive Inflammation

The evidence in favor of an antioxidant effect of HBOT makes a case for its use in preconditioning tissues to better resist ischemia and subsequent reperfusion injury. Although this is not an option in traumatic injury, the rapid introduction of the treatment after injury is still likely to reduce oxidative stress during reperfusion.

HBOT has been reported to have a beneficial effect on reperfusion injury, which may seem counterintuitive as the additional availability of oxygen supposedly increases oxidative stress at the injury site. HBOT may mitigate reperfusion injury through several mechanisms [139]. If HBOT is applied in a timely fashion, the additional oxygen is thought to maintain the viability of the marginal tissue, thus reducing cell death induced by the ischemia itself [155]. In a model of a tourniquet rat hind limb, HBOT was shown to preserve higher ATP levels, diminish the loss of the antioxidant glutathione after reperfusion, and decrease post-reperfusion edema [144]. Early HBOT may also suppress the progression of apoptosis, as seen in a model of traumatic spinal cord injury in a rat [152].

Another possible mechanism through which HBOT might counter the deleterious effects of reperfusion is by altering an excessive inflammatory response following reperfusion, as the therapy has been reported to have immunomodulatory effects in a variety of pro-inflammatory settings [139]. In an intestinal ischemia-reperfusion model, HBOT significantly reduced neutrophil recruitment and activation [156]. Similarly, it was found that HBOT reduces reperfusion injury in an in vitro endothelial cell model via the inhibition of ICAM-1 expression and integrin β2, possibly through the induction of endothelial nitric oxide synthase [157,158,159]. Numerous pro-inflammatory factors are decreased by HBOT, including nuclear transcription factor κB, IL-1β, IL-6, IL-8, TNFα, interferon γ, and platelet-activating factor [160,161,162,163,164,165,166,167,168,169,170,171].

### 5.3. Relative Hypoxia after HBOT and Angiogenic Effect

Another explanation of how HBOT might exercise its effect is by inducing relative hypoxia directly after the treatment. The transitory increase in oxygenation, followed by the normoxia (i.e., sensed as a relative hypoxia by cells) at the end of the treatment, could have a supportive effect on peripheral nerve regeneration. Indeed, repeated brief ischemia in a rat model of experimental sciatic nerve transection injury had a beneficial role in peripheral nerve regeneration [102]. Of particular relevance for this concept is a recent study reporting that acute intermittent hypoxia (11% O_2_ in breathing air of rats) improved the regeneration of surgically repaired peripheral nerves, namely enhancing myelination, increasing the numbers of newly myelinated fibers, and accelerating the return of toe spread function 5–10 weeks post-repair [172]. This concept was substantiated by the fact that numerous studies have found an increase in pro-angiogenic, hypoxia-inducible signals, such as HIF1α and VEGF, in other regenerative processes like cutaneous wound repair following HBOT [173]. In our laboratory, we found that HBOT led to a significantly accelerated reperfusion of ischemic cutaneous wounds in rats [140].

### 5.4. Pro-Regenerative Effects of HBOT

HBOT is likely to exert a beneficial effect on peripheral nerve regeneration by promoting pro-regenerative signaling cascades. Data specifically investigating the effect of HBOT on growth factor release during peripheral nerve regeneration are scarce, but the results from other fields of tissue regeneration advocate a pro-regenerative effect of HBOT on the growth factor landscape.

In a model of transient central nervous system ischemia, HBOT significantly reduced neurological injury in the ischemic injured cortex and decreased the levels of Nogo-A and reticulon 4 receptor, the latter being a key player in axonal growth inhibition [174]. Yang et al. demonstrated that HBOT treatment significantly reduced the level of the ischemia-induced downregulation of the important neurotrophic factor NT-3 mRNA and significantly increased cell survival after reperfusion in a model of forebrain ischemia [175]. In a traumatic murine brain model, HBOT significantly reduced the expression of apoptosis-promoting genes, such as c-fos, c-jun, and Bax, and weakened the activation of Caspase-3 in rat models [154]. A study on the effect of HBOT on erectile function recovery in a rat cavernous nerve injury model found that improved erective function was associated with higher NGF expression levels [176].

**Table 2:** Suggested effects on the redox environment of HBOT in peripheral nerve regeneration.

Mechanism of Action	Reported Redox-Related Effects	References
Antioxidant induction	Decrease in overall Nox activity and levels of oxidative stress	[153]
	Higher glutathione peroxidase production	[144,145]
	Increased activity of superoxide dismutase and catalase and decreased lipid peroxidation levels	[146]
	Upregulation of IL-10, BCL-2, heme oxygenase-1, and heat-shock proteins 70 and 72	[147,148,149,150,151,152]
	Inhibition of inducible and neuronal nitric oxide synthase	[147,148,149,150,151,152]
	NGF-mediated protection against oxidative stress via HO-1	[48,49,154]
Ischemia-reperfusion injury and modulation of inflammation	Reducing cell death during ischemia	[155]
	Reduced post-perfusion edema, higher post-perfusion glutathione level	[144]
	Reduced neutrophil recruitment and activation	[156]
	Reduced reperfusion injury via inhibition of ICAM-1 expression and integrin β2	[157,158,159]
	Inhibition of pro-inflammatory factors, including nuclear transcription factor κB, IL-1, IL-6, IL-8, TNF-α, interferon γ, and platelet-activating factor	[160,161,162,163,164,165,166,167,168,169,170,171]
Relative hypoxia, induction of neoangenenesis	Increases in HIF-1α, VEGFA, SDF-1, VEGFR2, and CXCR4	[173]
	Nox4/Nox2/pSer36-p66Shc feed-forward loop, providing yet another compelling example of the interplay	[94]
Pro-regenerative effects	Decreased Nogo-A and reticulon receptors	[174]
	Increased neurotrophin-3	[175]
	Decrease in apoptosis-promoting genes c-fos, c-jun, Ba, and Caspase-3	[154]
	Higher NGF expression	[176]
	Higher expression of neurotrophic factors NGF, BDNF, GDNF, and NF-3	[154]

### 5.5. Evidence of the Efficacy of HBOT in Improving Functional Outcome in Peripheral Nerve Injury

More than 20 years ago, in a sciatic nerve crush model in rabbits, Bradshaw et al. found that the morphological analysis of nerve specimens in the HBOT group at week 7 after injury resembled normal uncrushed nerves, with nerve fibers being uniformly distributed throughout the section, and myelination was similar to normal nerves, while untreated nerves showed edema and contained disarrayed nerve fibers [177]. Similarly, Haapaniemi et al. concluded that HBOT stimulates axonal outgrowth in a nerve crush model in rats [178]. In an axonal outgrowth graft model, outgrowth was significantly longer in animals that were treated with HBOT [179].

However, there are also some studies that did not find a significant functional benefit of HBOT regarding peripheral nerve regeneration. For instance, one study found that HBOT was not effective in the restoration of gait or muscular strength after 90 days in nerve-injured rats included in a crush injury model and a nerve transection and repair model [180]. In another rat sciatic crush injury model, a functional evaluation using a walking track analysis showed that HBOT did not have an effect on functional recovery [181]. In an acellular nerve and fresh cellular graft model evaluating Schwann cell migration and the invasion of macrophages 10 days after grafting, HBOT was not demonstrated to have an effect on the regeneration process in acellular nerve grafts. In contrast, the effect of HBOT on fresh cellular nerve grafts showed a significant improvement in axonal outgrowth and Schwann cell migration from the proximal nerve end [182].

A study using a model of sciatic nerve transection followed by microsurgical repair in rats showed mixed results. There was a statistical significance in conduction velocities and amplitude, and the foot/ankle angle showed a better response in the HBOT group compared to the untreated controls. Interestingly, the untreated group had higher numbers of axons and vessels at week 7, whereas there was no significant difference at week 14. Although there were more axons and myelin, the untreated animals seemed to be less functional than in the HBOT group, and the authors concluded that HBOT could improve functional recovery in this model [183]. In a model of guided neural regeneration with autologous fat grafting in a polyethylene tube associated with HBOT, the authors showed positive results on the morphometric and functional parameters of HBOT using Catwalk XT^®^ software (www.noldus.com) [184]. In transected, devascularized sciatic nerves following microsurgical repair, HBOT improved nerve function in a walking track analysis [185]. These mixed results highlight that there is still a lack of understanding as to what type of nerve injury benefits most from HBOT and that the effects might vary significantly depending on the experimental model. A recent review by Brenna et al. including 51 studies found that 88% of the studies reported HBOT to have a positive effect on peripheral nerve regeneration and/or resulted in a faster recovery time [186].

### 5.6. Conclusions and Clinical Relevance

The roles of oxygen in nerve regeneration range from energy supply, inflammation, and phagocytosis and crucial redox signaling cascades to oxidative cell destruction in the context of reperfusion injury. A fine balance between ROS production and antioxidant activity draws the line between physiological and pathological nerve regeneration. Some of the most compelling evidence on the key role of Nox-mediated redox signaling in nerve regeneration has been reported in recent years. This includes the internalization of macrophage-derived Nox2-containing exosomes into injured axons and its retrograde transportation into the cell nucleus where it induces regenerative outgrowth [22], which is a crucial role of Nox1/4 in Wallerian degeneration and of DUOX1 in axonal regeneration in cutaneous nerve injury [67]. This is a growing field, and the currently available data suggest that redox-modulating therapies have great therapeutic potential in the management of peripheral nerve lesions.

Thus, it is conceivable that increasing tissue oxygenation has a positive impact on peripheral nerve regeneration. One possibility to modulate the redox environment in injured nerves in clinics is hyperbaric oxygen. However, our current understanding of the mechanisms of action of HBOT in improving nerve regeneration remains limited. There is compelling evidence that HBOT beneficially modulates the redox environment, mainly from studies in tissues other than peripheral nerves. This includes the induction of antioxidants, the limitation of detrimental Nox-mediated ROS production during reperfusion-injury and the modulation of the inflammatory response, and the induction of relative hypoxia-inducing neoangiogenesis, as well as a pro-regenerative effect through redox-mediated growth factor activation.

HBOT seems to have a beneficial effect on functional recovery after peripheral nerve injury, but the results are mixed, suggesting that the treatment is efficient depending on the type of nerve defect and the experimental setup. More studies are required to confirm its efficacy and to better understand its mechanisms of action so as to better define indications and treatment protocols. Furthermore, the implication of Nox-mediated redox signaling in the key processes of peripheral nerve regeneration suggests that specific Nox-modulating drugs could influence regeneration capacities.

## Data Availability

The data are contained within the article. Additional data details will be provided upon request.

## References

[1] Scheib J., Hoke A. (2013). Advances in peripheral nerve regeneration. Nat. Rev. Neurol..

[2] Wood M.D., Kemp S.W., Weber C., Borschel G.H., Gordon T. (2011). Outcome measures of peripheral nerve regeneration. Ann. Anat..

[3] Hunt T.K., Zederfeldt B., Goldstick T.K. (1969). Oxygen and healing. Am. J. Surg..

[4] Tandara A.A., Mustoe T.A. (2004). Oxygen in wound healing--more than a nutrient. World J. Surg..

[5] Sen C.K., Roy S. (2008). Redox signals in wound healing. Biochim. Biophys. Acta.

[6] Bedard K., Krause K.H. (2007). The NOX family of ROS-generating NADPH oxidases: Physiology and pathophysiology. Physiol. Rev..

[7] Beckman K.B., Ames B.N. (1998). The free radical theory of aging matures. Physiol. Rev..

[8] Scott T.L., Rangaswamy S., Wicker C.A., Izumi T. (2014). Repair of oxidative DNA damage and cancer: Recent progress in DNA base excision repair. Antioxid. Redox Signal..

[9] Fukui K. (2016). Reactive oxygen species induce neurite degeneration before induction of cell death. J. Clin. Biochem. Nutr..

[10] Vincent A.M., Kato K., McLean L.L., Soules M.E., Feldman E.L. (2009). Sensory neurons and schwann cells respond to oxidative stress by increasing antioxidant defense mechanisms. Antioxid. Redox Signal..

[11] Inoguchi T., Sonta T., Tsubouchi H., Etoh T., Kakimoto M., Sonoda N., Sato N., Sekiguchi N., Kobayashi K., Sumimoto H. (2003). Protein kinase C-dependent increase in reactive oxygen species (ROS) production in vascular tissues of diabetes: Role of vascular NAD(P)H oxidase. J. Am. Soc. Nephrol. JASN.

[12] Sies H., Jones D.P. (2020). Reactive oxygen species (ROS) as pleiotropic physiological signalling agents. Nat. Rev. Mol. Cell Biol..

[13] D’Autreaux B., Toledano M.B. (2007). ROS as signalling molecules: Mechanisms that generate specificity in ROS homeostasis. Nat. Rev. Mol. Cell Biol..

[14] Holmstrom K.M., Finkel T. (2014). Cellular mechanisms and physiological consequences of redox-dependent signalling. Nat. Rev. Mol. Cell Biol..

[15] Spencer N.Y., Engelhardt J.F. (2014). The basic biology of redoxosomes in cytokine-mediated signal transduction and implications for disease-specific therapies. Biochemistry.

[16] Roy S., Khanna S., Nallu K., Hunt T.K., Sen C.K. (2006). Dermal wound healing is subject to redox control. Mol. Ther. J. Am. Soc. Gene Ther..

[17] Sen C.K. (2003). The general case for redox control of wound repair. Wound Repair Regen. Off. Publ. Wound Heal. Soc. Eur. Tissue Repair Soc..

[18] Andre-Levigne D., Modarressi A., Pepper M.S., Pittet-Cuenod B. (2017). Reactive Oxygen Species and NOX Enzymes Are Emerging as Key Players in Cutaneous Wound Repair. Int. J. Mol. Sci..

[19] Rieger S., Sagasti A. (2011). Hydrogen peroxide promotes injury-induced peripheral sensory axon regeneration in the zebrafish skin. PLoS Biol..

[20] Min J.Y., Park M.H., Park M.K., Park K.W., Lee N.W., Kim T., Kim H.J., Lee D.H. (2006). Staurosporin induces neurite outgrowth through ROS generation in HN33 hippocampal cell lines. J. Neural Transm..

[21] Pirotte N., Stevens A.S., Fraguas S., Plusquin M., Van Roten A., Van Belleghem F., Paesen R., Ameloot M., Cebria F., Artois T. (2015). Reactive Oxygen Species in Planarian Regeneration: An Upstream Necessity for Correct Patterning and Brain Formation. Oxid. Med. Cell. Longev..

[22] Hervera A., De Virgiliis F., Palmisano I., Zhou L., Tantardini E., Kong G., Hutson T., Danzi M.C., Perry R.B., Santos C.X.C. (2018). Reactive oxygen species regulate axonal regeneration through the release of exosomal NADPH oxidase 2 complexes into injured axons. Nat. Cell Biol..

[23] Eid S.A., Savelieff M.G., Eid A.A., Feldman E.L. (2021). Nox, Nox, Are You There? The Role of NADPH Oxidases in the Peripheral Nervous System. Antioxid. Redox Signal..

[24] Rishal I., Fainzilber M. (2014). Axon-soma communication in neuronal injury. Nat. Rev. Neurosci..

[25] Saito A., Cavalli V. (2016). Signaling Over Distances. Mol. Cell. Proteom..

[26] McKeown S.R. (2014). Defining normoxia, physoxia and hypoxia in tumours-implications for treatment response. Br. J. Radiol..

[27] Cai Q., Sheng Z.H. (2009). Mitochondrial transport and docking in axons. Exp. Neurol..

[28] Zhu X.H., Qiao H., Du F., Xiong Q., Liu X., Zhang X., Ugurbil K., Chen W. (2012). Quantitative imaging of energy expenditure in human brain. Neuroimage.

[29] Sasaki Y. (2019). Metabolic aspects of neuronal degeneration: From a NAD(+) point of view. Neurosci. Res..

[30] Vervust W., Ghysels A. (2022). Oxygen Storage in Stacked Phospholipid Membranes Under an Oxygen Gradient as a Model for Myelin Sheaths. Adv. Exp. Med. Biol..

[31] Nayernia Z., Jaquet V., Krause K.H. (2014). New insights on NOX enzymes in the central nervous system. Antioxid. Redox Signal..

[32] De Logu F., Nassini R., Materazzi S., Carvalho Goncalves M., Nosi D., Rossi Degl’Innocenti D., Marone I.M., Ferreira J., Li Puma S., Benemei S. (2017). Schwann cell TRPA1 mediates neuroinflammation that sustains macrophage-dependent neuropathic pain in mice. Nat. Commun..

[33] Kallenborn-Gerhardt W., Schroder K., Geisslinger G., Schmidtko A. (2013). NOXious signaling in pain processing. Pharmacol. Ther..

[34] Ibi M., Matsuno K., Shiba D., Katsuyama M., Iwata K., Kakehi T., Nakagawa T., Sango K., Shirai Y., Yokoyama T. (2008). Reactive oxygen species derived from NOX1/NADPH oxidase enhance inflammatory pain. J. Neurosci..

[35] Vincent A.M., Hayes J.M., McLean L.L., Vivekanandan-Giri A., Pennathur S., Feldman E.L. (2009). Dyslipidemia-induced neuropathy in mice: The role of oxLDL/LOX-1. Diabetes.

[36] Cao X., Demel S.L., Quinn M.T., Galligan J.J., Kreulen D. (2009). Localization of NADPH oxidase in sympathetic and sensory ganglion neurons and perivascular nerve fibers. Auton. Neurosci..

[37] Puntambekar P., Mukherjea D., Jajoo S., Ramkumar V. (2005). Essential role of Rac1/NADPH oxidase in nerve growth factor induction of TRPV1 expression. J. Neurochem..

[38] Coyoy A., Valencia A., Guemez-Gamboa A., Moran J. (2008). Role of NADPH oxidase in the apoptotic death of cultured cerebellar granule neurons. Free Radic. Biol. Med..

[39] Tammariello S.P., Quinn M.T., Estus S. (2000). NADPH oxidase contributes directly to oxidative stress and apoptosis in nerve growth factor-deprived sympathetic neurons. J. Neurosci..

[40] Johnson J.A., Johnson D.A., Kraft A.D., Calkins M.J., Jakel R.J., Vargas M.R., Chen P.C. (2008). The Nrf2-ARE pathway: An indicator and modulator of oxidative stress in neurodegeneration. Ann. N. Y. Acad. Sci..

[41] Kratsovnik E., Bromberg Y., Sperling O., Zoref-Shani E. (2005). Oxidative stress activates transcription factor NF-kB-mediated protective signaling in primary rat neuronal cultures. J. Mol. Neurosci..

[42] Gamper N., Ooi L. (2015). Redox and nitric oxide-mediated regulation of sensory neuron ion channel function. Antioxid. Redox Signal..

[43] Eftekharpour E., Fernyhough P. (2022). Oxidative Stress and Mitochondrial Dysfunction Associated with Peripheral Neuropathy in Type 1 Diabetes. Antioxid. Redox Signal..

[44] Zajaczkowska R., Kocot-Kepska M., Leppert W., Wrzosek A., Mika J., Wordliczek J. (2019). Mechanisms of Chemotherapy-Induced Peripheral Neuropathy. Int. J. Mol. Sci..

[45] Hichor M., Sundaram V.K., Eid S.A., Abdel-Rassoul R., Petit P.X., Borderie D., Bastin J., Eid A.A., Manuel M., Grenier J. (2018). Liver X Receptor exerts a protective effect against the oxidative stress in the peripheral nerve. Sci. Rep..

[46] Kallenborn-Gerhardt W., Lu R., Syhr K.M., Heidler J., von Melchner H., Geisslinger G., Bangsow T., Schmidtko A. (2013). Antioxidant activity of sestrin 2 controls neuropathic pain after peripheral nerve injury. Antioxid. Redox Signal..

[47] Lv W., Deng B., Duan W., Li Y., Liu Y., Li Z., Xia W., Li C. (2018). Schwann Cell Plasticity is Regulated by a Weakened Intrinsic Antioxidant Defense System in Acute Peripheral Nerve Injury. Neuroscience.

[48] Salinas M., Diaz R., Abraham N.G., Ruiz de Galarreta C.M., Cuadrado A. (2003). Nerve growth factor protects against 6-hydroxydopamine-induced oxidative stress by increasing expression of heme oxygenase-1 in a phosphatidylinositol 3-kinase-dependent manner. J. Biol. Chem..

[49] Dai C., Xiong J., Wang Y., Shen J., Velkov T., Xiao X. (2020). Nerve Growth Factor Confers Neuroprotection against Colistin-Induced Peripheral Neurotoxicity. ACS Infect. Dis..

[50] Hor C.P., Fung W.Y., Ang H.A., Lim S.C., Kam L.Y., Sim S.W., Lim L.H., Choon W.Y., Wong J.W. (2018). Efficacy of Oral Mixed Tocotrienols in Diabetic Peripheral Neuropathy: A Randomized Clinical Trial. JAMA Neurol..

[51] Saklayen M.G., Yap J., Vallyathan V. (2010). Effect of month-long treatment with oral N-acetylcysteine on the oxidative stress and proteinuria in patients with diabetic nephropathy: A pilot study. J. Investig. Med..

[52] Elbatreek M.H., Pachado M.P., Cuadrado A., Jandeleit-Dahm K., Schmidt H. (2019). Reactive Oxygen Comes of Age: Mechanism-Based Therapy of Diabetic End-Organ Damage. Trends Endocrinol. Metab..

[53] McElroy T., Zeidan R.S., Rathor L., Han S.M., Xiao R. (2023). The role of mitochondria in the recovery of neurons after injury. Neural Regen. Res..

[54] Clapham D.E. (2007). Calcium signaling. Cell.

[55] Giorgi C., Agnoletto C., Bononi A., Bonora M., De Marchi E., Marchi S., Missiroli S., Patergnani S., Poletti F., Rimessi A. (2012). Mitochondrial calcium homeostasis as potential target for mitochondrial medicine. Mitochondrion.

[56] Han S.M., Baig H.S., Hammarlund M. (2016). Mitochondria Localize to Injured Axons to Support Regeneration. Neuron.

[57] Zhou B., Yu P., Lin M.Y., Sun T., Chen Y., Sheng Z.H. (2016). Facilitation of axon regeneration by enhancing mitochondrial transport and rescuing energy deficits. J. Cell Biol..

[58] Horvath R., Medina J., Reilly M.M., Shy M.E., Zuchner S. (2023). Peripheral neuropathy in mitochondrial disease. Handb. Clin. Neurol..

[59] Gold R., Archelos J.J., Hartung H.P. (1999). Mechanisms of immune regulation in the peripheral nervous system. Brain Pathol..

[60] Giridharan S.S., Caplan S. (2014). MICAL-family proteins: Complex regulators of the actin cytoskeleton. Antioxid. Redox Signal..

[61] Kim M., Kim H., Kim D., Kim D., Huh Y., Park C., Chung H.J., Jung J., Jeong N.Y. (2019). Heme Oxygenase 1 in Schwann Cells Regulates Peripheral Nerve Degeneration Against Oxidative Stress. ASN Neuro.

[62] Feldman E.L., Callaghan B.C., Pop-Busui R., Zochodne D.W., Wright D.E., Bennett D.L., Bril V., Russell J.W., Viswanathan V. (2019). Diabetic neuropathy. Nat. Rev. Dis. Primers.

[63] Viader A., Sasaki Y., Kim S., Strickland A., Workman C.S., Yang K., Gross R.W., Milbrandt J. (2013). Aberrant Schwann cell lipid metabolism linked to mitochondrial deficits leads to axon degeneration and neuropathy. Neuron.

[64] Meda F., Gauron C., Rampon C., Teillon J., Volovitch M., Vriz S. (2016). Nerves Control Redox Levels in Mature Tissues Through Schwann Cells and Hedgehog Signaling. Antioxid. Redox Signal..

[65] Babetto E., Wong K.M., Beirowski B. (2020). A glycolytic shift in Schwann cells supports injured axons. Nat. Neurosci..

[66] Kallenborn-Gerhardt W., Schroder K., Del Turco D., Lu R., Kynast K., Kosowski J., Niederberger E., Shah A.M., Brandes R.P., Geisslinger G. (2012). NADPH oxidase-4 maintains neuropathic pain after peripheral nerve injury. J. Neurosci..

[67] Cadiz Diaz A., Schmidt N.A., Yamazaki M., Hsieh C.J., Lisse T.S., Rieger S. (2022). Coordinated NADPH oxidase/hydrogen peroxide functions regulate cutaneous sensory axon de- and regeneration. Proc. Natl. Acad. Sci. USA.

[68] Schafer M., Werner S. (2008). Oxidative stress in normal and impaired wound repair. Pharmacol. Res. Off. J. Ital. Pharmacol. Soc..

[69] Brooker R.J. (2012). Genetics: Analysis & Principles.

[70] Eckert J.W., Abramson S.L., Starke J., Brandt M.L. (1995). The surgical implications of chronic granulomatous disease. Am. J. Surg..

[71] Deffert C., Cachat J., Krause K.H. (2014). Phagocyte NADPH oxidase, chronic granulomatous disease and mycobacterial infections. Cell. Microbiol..

[72] Paulsen C.E., Carroll K.S. (2013). Cysteine-mediated redox signaling: Chemistry, biology, and tools for discovery. Chem. Rev..

[73] de Oliveira-Marques V., Cyrne L., Marinho H.S., Antunes F. (2007). A quantitative study of NF-kappaB activation by H_2_O_2_: Relevance in inflammation and synergy with TNF-alpha. J. Immunol..

[74] Klyubin I.V., Kirpichnikova K.M., Gamaley I.A. (1996). Hydrogen peroxide-induced chemotaxis of mouse peritoneal neutrophils. Eur. J. Cell Biol..

[75] Nakamura H., Herzenberg L.A., Bai J., Araya S., Kondo N., Nishinaka Y., Herzenberg L.A., Yodoi J. (2001). Circulating thioredoxin suppresses lipopolysaccharide-induced neutrophil chemotaxis. Proc. Natl. Acad. Sci. USA.

[76] Fraticelli A., Serrano C.V., Bochner B.S., Capogrossi M.C., Zweier J.L. (1996). Hydrogen peroxide and superoxide modulate leukocyte adhesion molecule expression and leukocyte endothelial adhesion. Biochim. Biophys. Acta.

[77] Ogura M., Kitamura M. (1998). Oxidant stress incites spreading of macrophages via extracellular signal-regulated kinases and p38 mitogen-activated protein kinase. J. Immunol..

[78] Lu H., Youker K., Ballantyne C., Entman M., Smith C.W. (2000). Hydrogen peroxide induces LFA-1-dependent neutrophil adherence to cardiac myocytes. Am. J. Physiol. Heart Circ. Physiol..

[79] Haddad J.J., Saade N.E., Safieh-Garabedian B. (2002). Redox regulation of TNF-alpha biosynthesis: Augmentation by irreversible inhibition of gamma-glutamylcysteine synthetase and the involvement of an IkappaB-alpha/NF-kappaB-independent pathway in alveolar epithelial cells. Cell. Signal..

[80] Lee H.K., Wang L., Shin Y.K., Lee K.Y., Suh D.J., Park H.T. (2009). Interleukin-6 induces proinflammatory signaling in Schwann cells: A high-throughput analysis. Biochem. Biophys. Res. Commun..

[81] Li B., Bedard K., Sorce S., Hinz B., Dubois-Dauphin M., Krause K.H. (2009). NOX4 expression in human microglia leads to constitutive generation of reactive oxygen species and to constitutive IL-6 expression. J. Innate Immun..

[82] Vilhardt F., Haslund-Vinding J., Jaquet V., McBean G. (2017). Microglia antioxidant systems and redox signalling. Br. J. Pharmacol..

[83] Li J., Lan T., Zhang C., Zeng C., Hou J., Yang Z., Zhang M., Liu J., Liu B. (2015). Reciprocal activation between IL-6/STAT3 and NOX4/Akt signalings promotes proliferation and survival of non-small cell lung cancer cells. Oncotarget.

[84] Eid S.A., El Massry M., Hichor M., Haddad M., Grenier J., Dia B., Barakat R., Boutary S., Chanal J., Aractingi S. (2020). Targeting the NADPH Oxidase-4 and Liver X Receptor Pathway Preserves Schwann Cell Integrity in Diabetic Mice. Diabetes.

[85] Levigne D., Modarressi A., Krause K.H., Pittet-Cuenod B. (2016). NADPH oxidase 4 deficiency leads to impaired wound repair and reduced dityrosine-crosslinking, but does not affect myofibroblast formation. Free Radic. Biol. Med..

[86] Le Belle J.E., Orozco N.M., Paucar A.A., Saxe J.P., Mottahedeh J., Pyle A.D., Wu H., Kornblum H.I. (2011). Proliferative neural stem cells have high endogenous ROS levels that regulate self-renewal and neurogenesis in a PI3K/Akt-dependant manner. Cell Stem Cell.

[87] De Virgiliis F., Hutson T.H., Palmisano I., Amachree S., Miao J., Zhou L., Todorova R., Thompson R., Danzi M.C., Lemmon V.P. (2020). Enriched conditioning expands the regenerative ability of sensory neurons after spinal cord injury via neuronal intrinsic redox signaling. Nat. Commun..

[88] Ye X., Qiu Y., Gao Y., Wan D., Zhu H. (2019). A Subtle Network Mediating Axon Guidance: Intrinsic Dynamic Structure of Growth Cone, Attractive and Repulsive Molecular Cues, and the Intermediate Role of Signaling Pathways. Neural Plast..

[89] Suzukawa K., Miura K., Mitsushita J., Resau J., Hirose K., Crystal R., Kamata T. (2000). Nerve growth factor-induced neuronal differentiation requires generation of Rac1-regulated reactive oxygen species. J. Biol. Chem..

[90] Kim D.S., An J.M., Lee H.G., Seo S.R., Kim S.S., Kim J.Y., Kang J.W., Bae Y.S., Seo J.T. (2013). Activation of Rac1-dependent redox signaling is critically involved in staurosporine-induced neurite outgrowth in PC12 cells. Free Radic. Res..

[91] Ibi M., Katsuyama M., Fan C., Iwata K., Nishinaka T., Yokoyama T., Yabe-Nishimura C. (2006). NOX1/NADPH oxidase negatively regulates nerve growth factor-induced neurite outgrowth. Free Radic. Biol. Med..

[92] Munnamalai V., Weaver C.J., Weisheit C.E., Venkatraman P., Agim Z.S., Quinn M.T., Suter D.M. (2014). Bidirectional interactions between NOX_2_-type NADPH oxidase and the F-actin cytoskeleton in neuronal growth cones. J. Neurochem..

[93] Feldman E.L., Nave K.A., Jensen T.S., Bennett D.L.H. (2017). New Horizons in Diabetic Neuropathy: Mechanisms, Bioenergetics, and Pain. Neuron.

[94] Montezano A.C., Burger D., Ceravolo G.S., Yusuf H., Montero M., Touyz R.M. (2011). Novel Nox homologues in the vasculature: Focusing on Nox_4_ and Nox_5_. Clin. Sci..

[95] Burger D., Montezano A.C., Nishigaki N., He Y., Carter A., Touyz R.M. (2011). Endothelial microparticle formation by angiotensin II is mediated via Ang II receptor type I/NADPH oxidase/ Rho kinase pathways targeted to lipid rafts. Arterioscler. Thromb. Vasc. Biol..

[96] Mezentsev A., Merks R.M., O’Riordan E., Chen J., Mendelev N., Goligorsky M.S., Brodsky S.V. (2005). Endothelial microparticles affect angiogenesis in vitro: Role of oxidative stress. Am. J. Physiol. Heart Circ. Physiol..

[97] Tripathi D., Biswas B., Manhas A., Singh A., Goyal D., Gaestel M., Jagavelu K. (2019). Proinflammatory Effect of Endothelial Microparticles Is Mitochondria Mediated and Modulated Through MAPKAPK2 (MAPK-Activated Protein Kinase 2) Leading to Attenuation of Cardiac Hypertrophy. Arterioscler. Thromb. Vasc. Biol..

[98] Burger D., Turner M., Munkonda M.N., Touyz R.M. (2016). Endothelial Microparticle-Derived Reactive Oxygen Species: Role in Endothelial Signaling and Vascular Function. Oxid. Med. Cell. Longev..

[99] Jansen F., Yang X., Franklin B.S., Hoelscher M., Schmitz T., Bedorf J., Nickenig G., Werner N. (2013). High glucose condition increases NADPH oxidase activity in endothelial microparticles that promote vascular inflammation. Cardiovasc. Res..

[100] Burnett M.G., Zager E.L. (2004). Pathophysiology of peripheral nerve injury: A brief review. Neurosurg. Focus.

[101] Smaila B.D., Holland S.D., Babaeijandaghi F., Henderson H.G., Rossi F.M.V., Ramer M.S. (2020). Systemic hypoxia mimicry enhances axonal regeneration and functional recovery following peripheral nerve injury. Exp. Neurol..

[102] Zhou X.B., Zou D.X., Gu W., Wang D., Feng J.S., Wang J.Y., Zhou J.L. (2018). An Experimental Study on Repeated Brief Ischemia in Promoting Sciatic Nerve Repair and Regeneration in Rats. World Neurosurg..

[103] Kim Y.M., Kim S.J., Tatsunami R., Yamamura H., Fukai T., Ushio-Fukai M. (2017). ROS-induced ROS release orchestrated by Nox4, Nox2, and mitochondria in VEGF signaling and angiogenesis. Am. J. Physiol. Cell Physiol..

[104] Schmelzer J.D., Zochodne D.W., Low P.A. (1989). Ischemic and reperfusion injury of rat peripheral nerve. Proc. Natl. Acad. Sci. USA.

[105] Zollman P.J., Awad O., Schmelzer J.D., Low P.A. (1991). Effect of ischemia and reperfusion in vivo on energy metabolism of rat sciatic-tibial and caudal nerves. Exp. Neurol..

[106] Baba M., Nukada H., McMorran D., Takahashi K., Wada R., Yagihashi S. (2006). Prolonged ischemic conduction failure after reperfusion in diabetic nerve. Muscle Nerve.

[107] Levigne D., Tobalem M., Modarressi A., Pittet-Cuenod B. (2013). Hyperglycemia Increases Susceptibility to Ischemic Necrosis. BioMed Res. Int..

[108] Low P.A., Schmelzer J.D., Ward K.K. (1986). The effect of age on energy metabolism and resistance to ischaemic conduction failure in rat peripheral nerve. J. Physiol..

[109] Shanmugasundaram K., Nayak B.K., Friedrichs W.E., Kaushik D., Rodriguez R., Block K. (2017). NOX4 functions as a mitochondrial energetic sensor coupling cancer metabolic reprogramming to drug resistance. Nat. Commun..

[110] Sciarretta S., Zhai P., Shao D., Zablocki D., Nagarajan N., Terada L.S., Volpe M., Sadoshima J. (2013). Activation of NADPH oxidase 4 in the endoplasmic reticulum promotes cardiomyocyte autophagy and survival during energy stress through the protein kinase RNA-activated-like endoplasmic reticulum kinase/eukaryotic initiation factor 2alpha/activating transcription factor 4 pathway. Circ. Res..

[111] Gomez-Sanchez J.A., Carty L., Iruarrizaga-Lejarreta M., Palomo-Irigoyen M., Varela-Rey M., Griffith M., Hantke J., Macias-Camara N., Azkargorta M., Aurrekoetxea I. (2015). Schwann cell autophagy, myelinophagy, initiates myelin clearance from injured nerves. J. Cell Biol..

[112] Allen D.B., Maguire J.J., Mahdavian M., Wicke C., Marcocci L., Scheuenstuhl H., Chang M., Le A.X., Hopf H.W., Hunt T.K. (1997). Wound hypoxia and acidosis limit neutrophil bacterial killing mechanisms. Arch. Surg..

[113] Soneja A., Drews M., Malinski T. (2005). Role of nitric oxide, nitroxidative and oxidative stress in wound healing. Pharmacol. Rep. PR.

[114] Anderson G.M., Nukada H., McMorran P.D. (1997). Carbonyl histochemistry in rat reperfusion nerve injury. Brain Res..

[115] Nagamatsu M., Schmelzer J.D., Zollman P.J., Smithson I.L., Nickander K.K., Low P.A. (1996). Ischemic reperfusion causes lipid peroxidation and fiber degeneration. Muscle Nerve.

[116] Mitsui Y., Schmelzer J.D., Zollman P.J., Kihara M., Low P.A. (1999). Hypothermic neuroprotection of peripheral nerve of rats from ischaemia-reperfusion injury. Brain.

[117] Iida H., Schmelzer J.D., Schmeichel A.M., Wang Y., Low P.A. (2003). Peripheral nerve ischemia: Reperfusion injury and fiber regeneration. Exp. Neurol..

[118] Carden D.L., Granger D.N. (2000). Pathophysiology of ischaemia-reperfusion injury. J. Pathol..

[119] Raedschelders K., Ansley D.M., Chen D.D. (2012). The cellular and molecular origin of reactive oxygen species generation during myocardial ischemia and reperfusion. Pharmacol. Ther..

[120] Sanderson T.H., Reynolds C.A., Kumar R., Przyklenk K., Huttemann M. (2013). Molecular mechanisms of ischemia-reperfusion injury in brain: Pivotal role of the mitochondrial membrane potential in reactive oxygen species generation. Mol. Neurobiol..

[121] Granger D.N., Kvietys P.R. (2015). Reperfusion injury and reactive oxygen species: The evolution of a concept. Redox Biol..

[122] Simone S., Rascio F., Castellano G., Divella C., Chieti A., Ditonno P., Battaglia M., Crovace A., Staffieri F., Oortwijn B. (2014). Complement-dependent NADPH oxidase enzyme activation in renal ischemia/reperfusion injury. Free Radic. Biol. Med..

[123] Nakagiri A., Sunamoto M., Murakami M. (2007). NADPH oxidase is involved in ischaemia/reperfusion-induced damage in rat gastric mucosa via ROS production--role of NADPH oxidase in rat stomachs. Inflammopharmacology.

[124] Choi D.H., Kim J.H., Lee K.H., Kim H.Y., Kim Y.S., Choi W.S., Lee J. (2015). Role of neuronal NADPH oxidase 1 in the peri-infarct regions after stroke. PLoS ONE.

[125] Miller A.A., Dusting G.J., Roulston C.L., Sobey C.G. (2006). NADPH-oxidase activity is elevated in penumbral and non-ischemic cerebral arteries following stroke. Brain Res..

[126] Yokota H., Narayanan S.P., Zhang W., Liu H., Rojas M., Xu Z., Lemtalsi T., Nagaoka T., Yoshida A., Brooks S.E. (2011). Neuroprotection from retinal ischemia/reperfusion injury by NOX2 NADPH oxidase deletion. Investig. Ophthalmol. Vis. Sci..

[127] Doerries C., Grote K., Hilfiker-Kleiner D., Luchtefeld M., Schaefer A., Holland S.M., Sorrentino S., Manes C., Schieffer B., Drexler H. (2007). Critical role of the NAD(P)H oxidase subunit p47phox for left ventricular remodeling/dysfunction and survival after myocardial infarction. Circ. Res..

[128] Gan X., Su G., Zhao W., Huang P., Luo G., Hei Z. (2013). The mechanism of sevoflurane preconditioning-induced protections against small intestinal ischemia reperfusion injury is independent of mast cell in rats. Mediat. Inflamm..

[129] Chen H., Song Y.S., Chan P.H. (2009). Inhibition of NADPH oxidase is neuroprotective after ischemia-reperfusion. J. Cereb. Blood Flow Metab..

[130] Uysal A., Sahna E., Ozguler I.M., Burma O., Ilhan N. (2015). Effects of apocynin, an NADPH oxidase inhibitor, on levels of ADMA, MPO, iNOS and TLR4 induced by myocardial ischemia reperfusion. Perfusion.

[131] Lee I., Dodia C., Chatterjee S., Zagorski J., Mesaros C., Blair I.A., Feinstein S.I., Jain M., Fisher A.B. (2013). A novel nontoxic inhibitor of the activation of NADPH oxidase reduces reactive oxygen species production in mouse lung. J. Pharmacol. Exp. Ther..

[132] Paterniti I., Galuppo M., Mazzon E., Impellizzeri D., Esposito E., Bramanti P., Cuzzocrea S. (2010). Protective effects of apocynin, an inhibitor of NADPH oxidase activity, in splanchnic artery occlusion and reperfusion. J. Leukoc. Biol..

[133] Korthuis R.J., Gute D.C., Blecha F., Ross C.R. (1999). PR-39, a proline/arginine-rich antimicrobial peptide, prevents postischemic microvascular dysfunction. Am. J. Physiol..

[134] Braunersreuther V., Montecucco F., Asrih M., Pelli G., Galan K., Frias M., Burger F., Quindere A.L., Montessuit C., Krause K.H. (2013). Role of NADPH oxidase isoforms NOX1, NOX2 and NOX4 in myocardial ischemia/reperfusion injury. J. Mol. Cell. Cardiol..

[135] Zhang Y., Wang T., Yang K., Xu J., Wu J.M., Liu W.L. (2016). NADPH oxidase 2 does not contribute to early reperfusion-associated reactive oxygen species generation following transient focal cerebral ischemia. Neural Regen. Res..

[136] Urner S., Ho F., Jha J.C., Ziegler D., Jandeleit-Dahm K. (2020). NADPH Oxidase Inhibition: Preclinical and Clinical Studies in Diabetic Complications. Antioxid. Redox Signal..

[137] Carter M.J., Frykberg R.G., Oropallo A., Sen C.K., Armstrong D.G., Nair H.K.R., Serena T.E. (2023). Efficacy of Topical Wound Oxygen Therapy in Healing Chronic Diabetic Foot Ulcers: Systematic Review and Meta-Analysis. Adv. Wound Care.

[138] Sen C.K. (2009). Wound healing essentials: Let there be oxygen. Wound Repair Regen. Off. Publ. Wound Heal. Soc. Eur. Tissue Repair Soc..

[139] Sanchez E.C. (2007). Hyperbaric oxygenation in peripheral nerve repair and regeneration. Neurol. Res..

[140] Andre-Levigne D., Modarressi A., Pignel R., Bochaton-Piallat M.L., Pittet-Cuenod B. (2016). Hyperbaric oxygen therapy promotes wound repair in ischemic and hyperglycemic conditions, increasing tissue perfusion and collagen deposition. Wound Repair Regen. Off. Publ. Wound Heal. Soc. Eur. Tissue Repair Soc..

[141] Nazario J., Kuffler D.P. (2011). Hyperbaric oxygen therapy and promoting neurological recovery following nerve trauma. Undersea Hyperb. Med..

[142] Dong H., Xiong L., Zhu Z., Chen S., Hou L., Sakabe T. (2002). Preconditioning with hyperbaric oxygen and hyperoxia induces tolerance against spinal cord ischemia in rabbits. Anesthesiology.

[143] Nie H., Xiong L., Lao N., Chen S., Xu N., Zhu Z. (2006). Hyperbaric oxygen preconditioning induces tolerance against spinal cord ischemia by upregulation of antioxidant enzymes in rabbits. J. Cereb. Blood Flow Metab..

[144] Haapaniemi T., Sirsjo A., Nylander G., Larsson J. (1995). Hyperbaric oxygen treatment attenuates glutathione depletion and improves metabolic restitution in postischemic skeletal muscle. Free Radic. Res..

[145] Yasar M., Yildiz S., Mas R., Dundar K., Yildirim A., Korkmaz A., Akay C., Kaymakcioglu N., Ozisik T., Sen D. (2003). The effect of hyperbaric oxygen treatment on oxidative stress in experimental acute necrotizing pancreatitis. Physiol. Res..

[146] Shams Z., Khalatbary A.R., Ahmadvand H., Zare Z., Kian K. (2017). Neuroprotective effects of hyperbaric oxygen (HBO) therapy on neuronal death induced by sciatic nerve transection in rat. BMC Neurol..

[147] Kurata S., Yamashita U., Nakajima H. (1995). Hyperbaric oxygenation reduces the cytostatic activity and transcription of nitric oxide synthetase gene of mouse peritoneal macrophages. Biochim. Biophys. Acta.

[148] Rothfuss A., Speit G. (2002). Investigations on the mechanism of hyperbaric oxygen (HBO)-induced adaptive protection against oxidative stress. Mutat. Res..

[149] Wada K., Miyazawa T., Nomura N., Tsuzuki N., Nawashiro H., Shima K. (2001). Preferential conditions for and possible mechanisms of induction of ischemic tolerance by repeated hyperbaric oxygenation in gerbil hippocampus. Neurosurgery.

[150] Rosenthal R.E., Silbergleit R., Hof P.R., Haywood Y., Fiskum G. (2003). Hyperbaric oxygen reduces neuronal death and improves neurological outcome after canine cardiac arrest. Stroke.

[151] Shyu W.C., Lin S.Z., Saeki K., Kubosaki A., Matsumoto Y., Onodera T., Chiang M.F., Thajeb P., Li H. (2004). Hyperbaric oxygen enhances the expression of prion protein and heat shock protein 70 in a mouse neuroblastoma cell line. Cell. Mol. Neurobiol..

[152] Yu Y., Matsuyama Y., Yanase M., Ito S., Adachi K., Satake K., Ishiguro N., Kiuchi K. (2004). Effects of hyperbaric oxygen on GDNF expression and apoptosis in spinal cord injury. Neuroreport.

[153] Ostrowski R.P., Tang J., Zhang J.H. (2006). Hyperbaric oxygen suppresses NADPH oxidase in a rat subarachnoid hemorrhage model. Stroke.

[154] Xing P., Ma K., Li L., Wang D., Hu G., Long W. (2018). The protection effect and mechanism of hyperbaric oxygen therapy in rat brain with traumatic injury. Acta Cir. Bras..

[155] Garcia-Covarrubias L., Cuauhtemoc Sanchez-Rodriguez E. (2000). Hyperbaric oxygenation therapy, basic concepts. Gac. Med. Mex..

[156] Tjarnstrom J., Wikstrom T., Bagge U., Risberg B., Braide M. (1999). Effects of hyperbaric oxygen treatment on neutrophil activation and pulmonary sequestration in intestinal ischemia-reperfusion in rats. Eur. Surg. Res..

[157] Buras J.A., Stahl G.L., Svoboda K.K., Reenstra W.R. (2000). Hyperbaric oxygen downregulates ICAM-1 expression induced by hypoxia and hypoglycemia: The role of NOS. Am. J. Physiol. Cell Physiol..

[158] Chen Q., Banick P.D., Thom S.R. (1996). Functional inhibition of rat polymorphonuclear leukocyte B2 integrins by hyperbaric oxygen is associated with impaired cGMP synthesis. J. Pharmacol. Exp. Ther..

[159] Thom S.R., Mendiguren I., Hardy K., Bolotin T., Fisher D., Nebolon M., Kilpatrick L. (1997). Inhibition of human neutrophil beta2-integrin-dependent adherence by hyperbaric O_2_. Am. J. Physiol..

[160] Sakoda M., Ueno S., Kihara K., Arikawa K., Dogomori H., Nuruki K., Takao S., Aikou T. (2004). A potential role of hyperbaric oxygen exposure through intestinal nuclear factor-kappaB. Crit. Care Med..

[161] Weisz G., Lavy A., Adir Y., Melamed Y., Rubin D., Eidelman S., Pollack S. (1997). Modification of in vivo and in vitro TNF-alpha, IL-1, and IL-6 secretion by circulating monocytes during hyperbaric oxygen treatment in patients with perianal Crohn’s disease. J. Clin. Immunol..

[162] Bitterman N., Bitterman H., Kinarty A., Melamed Y., Lahat N. (1993). Effect of a single exposure to hyperbaric oxygen on blood mononuclear cells in human subjects. Undersea Hyperb. Med..

[163] Inamoto Y., Okuno F., Saito K., Tanaka Y., Watanabe K., Morimoto I., Yamashita U., Eto S. (1991). Effect of hyperbaric oxygenation on macrophage function in mice. Biochem. Biophys. Res. Commun..

[164] Yamashita M., Yamashita M. (2000). Hyperbaric oxygen treatment attenuates cytokine induction after massive hemorrhage. Am. J. Physiol. Endocrinol. Metab..

[165] Benson R.M., Minter L.M., Osborne B.A., Granowitz E.V. (2003). Hyperbaric oxygen inhibits stimulus-induced proinflammatory cytokine synthesis by human blood-derived monocyte-macrophages. Clin. Exp. Immunol..

[166] Rocco M., Antonelli M., Letizia V., Alampi D., Spadetta G., Passariello M., Conti G., Serio P., Gasparetto A. (2001). Lipid peroxidation, circulating cytokine and endothelin 1 levels in healthy volunteers undergoing hyperbaric oxygenation. Minerva Anestesiol..

[167] Granowitz E.V., Skulsky E.J., Benson R.M., Wright J., Garb J.L., Cohen E.R., Smithline E.C., Brown R.B. (2002). Exposure to increased pressure or hyperbaric oxygen suppresses interferon-gamma secretion in whole blood cultures of healthy humans. Undersea Hyperb. Med..

[168] Yang Z.J., Bosco G., Montante A., Ou X.I., Camporesi E.M. (2001). Hyperbaric O_2_ reduces intestinal ischemia-reperfusion-induced TNF-alpha production and lung neutrophil sequestration. Eur. J. Appl. Physiol..

[169] van den Blink B., van der Kleij A.J., Versteeg H.H., Peppelenbosch M.P. (2002). Immunomodulatory effect of oxygen and pressure. Comp. Biochem. Physiol. A Mol. Integr. Physiol..

[170] Lin H.C., Wan F.J., Wu C.C., Tung C.S., Wu T.H. (2005). Hyperbaric oxygen protects against lipopolysaccharide-stimulated oxidative stress and mortality in rats. Eur. J. Pharmacol..

[171] Tsai H.M., Gao C.J., Li W.X., Lin M.T., Niu K.C. (2005). Resuscitation from experimental heatstroke by hyperbaric oxygen therapy. Crit. Care Med..

[172] Nadeau J.R., Arnold B.M., Johnston J.M., Muir G.D., Verge V.M.K. (2021). Acute intermittent hypoxia enhances regeneration of surgically repaired peripheral nerves in a manner akin to electrical stimulation. Exp. Neurol..

[173] Huang X., Liang P., Jiang B., Zhang P., Yu W., Duan M., Guo L., Cui X., Huang M., Huang X. (2020). Hyperbaric oxygen potentiates diabetic wound healing by promoting fibroblast cell proliferation and endothelial cell angiogenesis. Life Sci..

[174] Zhou C., Li Y., Nanda A., Zhang J.H. (2003). HBO suppresses Nogo-A, Ng-R, or RhoA expression in the cerebral cortex after global ischemia. Biochem. Biophys. Res. Commun..

[175] Yang J.T., Chang C.N., Lee T.H., Lin T.N., Hsu J.C., Hsu Y.H., Wu J.H. (2001). Hyperbaric oxygen treatment decreases post-ischemic neurotrophin-3 mRNA down-regulation in the rat hippocampus. Neuroreport.

[176] Muller A., Tal R., Donohue J.F., Akin-Olugbade Y., Kobylarz K., Paduch D., Cutter S.C., Mehrara B.J., Scardino P.T., Mulhall J.P. (2008). The effect of hyperbaric oxygen therapy on erectile function recovery in a rat cavernous nerve injury model. J. Sex. Med..

[177] Bradshaw P.O., Nelson A.G., Fanton J.W., Yates T., Kagan-Hallet K.S. (1996). Effect of hyperbaric oxygenation on peripheral nerve regeneration in adult male rabbits. Undersea Hyperb. Med..

[178] Haapaniemi T., Nylander G., Kanje M., Dahlin L. (1998). Hyperbaric oxygen treatment enhances regeneration of the rat sciatic nerve. Exp. Neurol..

[179] Haapaniemi T., Nishiura Y., Dahlin L.B. (2001). Effects of hyperbaric oxygen treatment on axonal outgrowth in sciatic nerve grafts in rats. Scand. J. Plast. Reconstr. Surg. Hand Surg..

[180] Haapaniemi T., Nishiura Y., Dahlin L.B. (2002). Functional evaluation after rat sciatic nerve injury followed by hyperbaric oxygen treatment. J. Peripher. Nerv. Syst..

[181] Tuma Junior P., Dias M.D., Arrunategui G., Duarte G.G., Wada A., Cunha A.S., Ferreira M.C. (1999). Effect of hyperbaric oxygen on the regeneration of experimental crush injuries of nerves. Rev. Hosp. Clin. Fac. Med. Sao Paulo.

[182] Nishiura Y., Haapaniemi T., Dahlin L.B. (2001). Hyperbaric oxygen treatment has different effects on nerve regeneration in acellular nerve and muscle grafts. J. Peripher. Nerv. Syst..

[183] Eguiluz-Ordonez R., Sanchez C.E., Venegas A., Figueroa-Granados V., Hernandez-Pando R. (2006). Effects of hyperbaric oxygen on peripheral nerves. Plast. Reconstr. Surg..

[184] Toledo G.L., Sangalette B.S., Passerotti L.C., Nascimento J.A., Shinohara A.L., Oliveira A.L.R., Buzalaf M.A.R., Rodrigues A.C. (2021). Guided neural regeneration with autologous fat grafting and oxygen hyperbaric therapy. Braz. Oral Res..

[185] Zamboni W.A., Brown R.E., Roth A.C., Mathur A., Stephenson L.L. (1995). Functional evaluation of peripheral-nerve repair and the effect of hyperbaric oxygen. J. Reconstr. Microsurg..

[186] Brenna C.T., Khan S., Katznelson R., Brull R. (2023). The role of hyperbaric oxygen therapy in the management of perioperative peripheral nerve injury: A scoping review of the literature. Reg. Anesth. Pain Med..

